# Potential suicide risk among the college student population: machine learning approaches for identifying predictors and different students’ risk profiles

**DOI:** 10.1186/s41155-024-00301-6

**Published:** 2024-05-17

**Authors:** Jessica Dagani, Chiara Buizza, Clarissa Ferrari, Alberto Ghilardi

**Affiliations:** 1 Department of Clinical and Experimental Sciences, Section of Clinical and Dynamic Psychology, University of Brescia, Viale Europa, 11, 25123 Brescia, Italy; 2 Istituto Ospedaliero Fondazione Poliambulanza, Via Bissolati, 57, 25124 Brescia, Italy

**Keywords:** Suicide, Students, Machine learning, Risk factors

## Abstract

**Background:**

Suicide is one of the leading causes of death among young people and university students. Research has identified numerous socio-demographic, relational, and clinical factors as potential predictors of suicide risk, and machine learning techniques have emerged as promising ways to improve risk assessment.

**Objective:**

This cross-sectional observational study aimed at identifying predictors and college student profiles associated with suicide risk through a machine learning approach.

**Methods:**

A total of 3102 students were surveyed regarding potential suicide risk, socio-demographic characteristics, academic career, and physical/mental health and well-being. The classification tree technique and the multiple correspondence analysis were applied to define students’ profiles in terms of suicide risk and to detect the main predictors of such a risk.

**Results:**

Among the participating students, 7% showed high potential suicide risk and 3.8% had a history of suicide attempts. Psychological distress and use of alcohol/substance were prominent predictors of suicide risk contributing to define the profile of high risk of suicide: students with significant psychological distress, and with medium/high-risk use of alcohol and psychoactive substances. Conversely, low psychological distress and low-risk use of alcohol and substances, together with religious practice, represented the profile of students with low risk of suicide.

**Conclusions:**

Machine learning techniques could hold promise for assessing suicide risk in college students, potentially leading to the development of more effective prevention programs. These programs should address both risk and protective factors and be tailored to students’ needs and to the different categories of risk.

**Supplementary Information:**

The online version contains supplementary material available at 10.1186/s41155-024-00301-6.

## Introduction

Suicide can be considered the most severe expression of psychological distress and is a serious public health concern, being ranked as a leading cause of death among people aged between 15 and 29 (World Health Organization, 2023). This age includes people’s college years and university is in itself a highly stressful environment (Deasy et al., 2014; Robotham & Julian, 2006), where students have to deal with academic pressure and lots of other stressful tasks; college students therefore present high rates of anxiety, depression, and other mental health problems (Blanco et al., 2008; Bruffaerts et al., 2019). Indeed, evidence has shown that prevalence estimates of suicidal behavior in college students are consistently high (Eskin et al., 2016; Mortier et al., 2018; O’Neill et al., 2018). In Italy, only a few studies have evaluated suicidal risk and behavior in college students (Eskin et al., 2016; Stefa-Missagli et al., 2020; Tarchi et al., 2021): available data report around 20% of students presenting a lifetime of suicidal ideation and nearly 3% with a history of previous suicide attempts (Eskin et al., 2016).

Suicide risk assessment and targeted interventions for groups at high suicide risk are currently key elements of suicide prevention. Risk factors for suicide have been investigated at both the environmental and individual levels, including lack of social support, life events, family history, early-life adversities, substance misuse, and mental health difficulties (Fazel & Runeson, 2020). Conversely, strong personal relationships, personal well-being, and religious or spiritual beliefs have been reported as protective factors (World Health Organization, 2014). College is not only a stressful experience for students; it also serves as a crucial setting where students’ suicidal ideation can be detected and analyzed, and where targeted preventive actions can be taken (Wolitzky-Taylor et al., 2020). For these reasons, suicidality in college students has drawn the attention of many researchers, who have highlighted a combination of specific individual, relational, and academic factors. These include non-heterosexual orientation (Assari, 2018; Mortier et al., 2018; O'Neill et al., 2018), drinking and drug use (Arria et al., 2009; Assari, 2018; Shen et al., 2020), high levels of psychological distress (Eskin et al., 2016; Garlow et al., 2008), medicine majors, extra years of schooling (Uchida & Uchida, 2017), and low academic performance (Bruffaerts et al., 2018) as risk factors. The practice of a religion was confirmed as a protective factor, reducing the odds of suicidal behavior (Assari, 2018).

The well-established understanding of suicide as a complex phenomenon underscores a critical challenge in risk assessment. While extensive research over the past five decades has identified numerous environmental, individual, and relational risk factors, traditional statistical techniques have yielded models with only marginally better than chance predictive power (Franklin et al., 2017). This limitation stems from the inherent constraints of these methods, which often restrict the number of variables analyzed simultaneously, hindering the development of nuanced and comprehensive predictive models.

Developing an efficient model able to predict individuals’ suicide risk would be an important step forward for suicide assessment. In order to overcome the limitations of traditional statistical approaches, machine learning methods emerged as convenient tools in different medical settings (Rajkomar et al., 2019) and have been used as effective screening and evaluation tools for suicide risk assessment (Shen et al., 2020). Machine learning techniques offer a significant advantage over traditional methods for suicide risk assessment: by enabling predictions at the individual level, they allow for a more nuanced understanding of the complex factors contributing to suicidality (Pigoni et al., 2024). Moreover, they enable the simultaneous testing of a multitude of factors and their complex interactions, and they are able to model subgroups of individuals (Burke et al., 2019). Recent studies applied machine learning techniques, including classification tree analysis and multiple correspondence analysis (MCA), to identify profiles of at-risk individuals in samples of adolescents (Hill et al., 2017; Méndez-Bustos et al., 2022) and adults (Baneshi et al., 2017). The results of these studies highlighted the potential of machine learning techniques for significantly improving the identification of the profile of individuals at risk for suicide. Although these techniques promise insightful investigation in psycho-social and epidemiology fields, it is well known that they are fully data-driven approaches and, for this reason, they need large amounts of data to ensure reliable and accurate results (Goldenholz et al., 2023). Similarly, the interpretability of machine learning findings could be hard if not supported by preliminary hypotheses or validated by further analytical methods and techniques (Carvalho et al., 2019). In such a context, the machine learning approach reveals its full effectiveness on sufficiently large dataset analyzed following well-established hypotheses and validating the findings by different methods.

In the present study, we evaluated the epidemiology of suicidality in a cohort of Italian college students, aiming to identify predictors and student profiles associated with suicide risk through a machine learning approach. The use of two different approaches applied to a large cohort will ensure reliable, robust, and interpretable results.

## Methods

### Procedures

For this cross-sectional observational study conducted at a medium-sized Italian university, a multidimensional online survey was implemented through LimeSurvey (www.limesurvey.org), an open-source software that allows for completely anonymous data collection, so that only de-identified data were delivered to the investigators in order to preserve participants’ anonymity. A detailed description of the study was sent via email to all students on the university’s student mailing list. This email included the link to access the online survey and, via this link, students were asked to confirm their consent to participate. The study was conducted in accordance with the World Medical Association’s Helsinki Declaration for Human Studies and organizational ethic approvals were obtained from the university’s institutional review board (approved with provision no. 56 on 27/09/2018 and then ratified in memorandum no. 11.1/2018 on 10/10/2018) before contacting students. The online survey was implemented following Pealer and Weiler’s guidelines (Pealer & Weiler, 2003) and using a number of strategies to maximize the response rate suggested by Edwards et al. (2009), including sending reminders. Indeed, every week (for a total of 6 weeks) an email was automatically sent to students who had not completed the survey, reminding them to participate or to complete it.

Discussions with students helped form the basis for survey content development, item selection, and survey implementation. The survey was piloted and further modified using a “convenience” group of students from other state universities, thereby avoiding contamination of the intended study sample. Recruitment and data collection took place between May and June 2019.

### Study sample

Out of the 13,886 students in the study population, 3754 (27.1%) agreed to participate by accessing the online survey and 3102 completed the suicide risk assessment. The mean age of participants was 23.09 years (SD = 4.69, median value = 22, interquartile range = 20–23). Most students were female (58%) and Italian (94.8%) and identified as heterosexual (91.3%). Most students were enrolled in a medicine major (38.5%), while the others were studying engineering (26.9%), economics (18.9%), and law (7.7%). Among the 2405 students who were devotees of a religion, 51.9% were Christian.

### Survey instruments

This multidimensional survey assessed a wide range of socio-demographic and academic characteristics. More specifically, the first section of the survey included questions on students’ personal life and beliefs, housing situation and daily routine, past and actual substance use, perceived quality of their health, and university experience in terms of academic career and achievements. Moreover, the survey incorporated several standardized instruments.

#### Suicide risk

The P4 Screener is a brief tool to assess potential suicide risk (Dube et al., 2010), which includes a pre-screening question about thoughts of self-harming (“Have you had thoughts of actually hurting yourself?”); if a positive answer is given to this pre-screening question, there are then subsequent questions on the “4 P’s”: Past suicide attempts, Plan, Probability of completing suicide, and Preventive factors. Potential suicide risk is classified as minimal, lower, or higher. There is considered to be a “Minimal” risk when there is no past history, no suicide plan, and a “not at all likely” probability of an attempt. “Lower” risk refers to respondents who indicated a plan and/or past history but responded “not at all likely” to the question regarding probability and noted there were factors preventing them from taking action. “Higher” risk respondents are those who reported the probability of a suicide attempt as being either “somewhat likely” or “very likely” and/or reported there were no factors preventing them from taking action. If respondents give a negative answer to the pre-screening question, they are classed in the “Did not trigger” category of risk.

#### Mental health and well-being

The General Health Questionnaire (GHQ-12) is a well-known, self-administered, symptom-based rating scale of mental health and psychophysical well-being (Goldberg & Blackwell, 1970). This screening tool comprises 12 questions investigating the presence and frequency of non-chronic symptoms over the previous 4 weeks. Each item has a 4-point response scale; in this study, we used the standard bi-modal method of scoring (0–0-1–1), in which a score of 0 was assigned to the first two low-stress alternatives and a score of 1 was given to the two high-stress alternatives. In line with several studies on college students (Guthrie et al., 1998; James et al., 2013a; Moffat et al., 2004), we chose a cut-off point of 3, above which there is an indication of significant psychological distress. The Italian version of the GHQ-12 showed good reliability, as indicated by a Cronbach’s alpha of 0.81 (Politi et al., 1994). It has already been used in the context of Italian college students, showing a Cronbach’s alpha value of 0.85 (Preti et al., 2013). In this sample, the overall Cronbach’s alpha value was 0.79.

#### Psychological distress

The University Stress Scale (USS) is a self-administered screening questionnaire including 21 items that capture the cognitive appraisal of demands across the range of environmental stressors experienced by students (Stallman & Hurst, 2016). Each item scores from 0 (“Not at all”) to 3 (“Constantly”), and the sum of all items gives the extent score, ranging from 0 to 63. An extent score equal to or above 13 is predictive of significant psychological distress. The USS showed good reliability as indicated by a Cronbach’s alpha of 0.83 (Stallman, 2008). In this sample, the overall Cronbach’s alpha value was confirmed at 0.83.

#### Substance use

Participants were asked to complete a modified version of the World Health Organization’s Alcohol, Smoking and Substance Involvement Screening Test v3.0 (ASSIST), a questionnaire based on a self-report adaptation of Barreto and colleagues, aimed at detecting and managing substance use (Barreto et al., 2014). It contains eight questions covering 10 substances: tobacco, alcohol, cannabis, cocaine, amphetamine-type stimulants, inhalants, sedatives, hallucinogens, opioids, and “other drugs.” A score was determined for each substance and categorized as low-, moderate-, or high-risk use. The ability of ASSIST to classify respondents based on their degree of drug use has been extensively validated (Humeniuk et al., 2008, 2012). In the original study, Cronbach’s alpha values ranged from 0.71 to 0.90 (Barreto et al., 2014). In a recent Italian large population study, the ASSIST showed Cronbach’s alpha values ranging from 0.70 and 0.81 (Aas et al., 2024), in line with our study in which the Cronbach’s alpha values ranged from 0.66 to 0.93.

As the survey responses were entirely anonymous to the investigators, there was no mechanism for providing students who reported high levels of distress or recent suicidal ideation with specific referral to mental health resources. However, students with P4 Screener scores that indicated a higher suicide risk were shown an automatic message on their screen, suggesting that they contact the university counseling service (we provided the phone and email contacts of the service), their general practitioner, or a mental health professional. At the same time, we improved the promotion of the university counseling service through the university website, in order to facilitate students’ access to proper care.

### Statistical analysis

Descriptive statistics were computed for socio-demographic and academic characteristics and for the questionnaire scores, with percentage distribution for categorical variables and mean and standard deviation (SD) for quantitative variables. Chi-square tests were applied to compare the risk of suicide (categorical variable defined by the P4 Screener tool) across categories of socio-demographic and academic variables, levels of substance use risk, and levels of psychological distress. The ANOVA test was used to compare the means of continuous variables across categories of suicide risk.

The decision tree technique for categorical outcome (i.e., the Classification Tree, or CT, method) was applied to detect the most important variables in predicting the main outcome, i.e., suicide risk. CTs allowed for homogeneous groups (student profiles) with different levels of suicide risk to be highlighted. The CT output was given by different pathways (defined by the estimated regressor cut-offs or categories) and, for each of them, the estimated predicted frequencies (percentages) of the dependent variable categories. The prediction accuracy of the CTs was evaluated through a confusion matrix with the percentage of correct classification (James et al., 2013b). Finally, in order to provide a further assessment of the students’ profiles in terms of suicide risk, a second machine learning approach for categorical variable was performed: the MCA. The outcome of this method was represented in a unique two-dimensional space plot (Biplot) showing the relationship between individuals (points) and the categorical variables. Variable categories that are in the same quadrant, or that are close enough, are considered to be in a mutual relationship and in association with the closest individual subgroups. This graphical representation of MCA output allows for specific subject profiles to be identified (Rencher, 2003).

All tests were two-tailed and the probability of a type I error was set at *p* < 0.05. The analyses were performed using IBM SPSS Statistics for Windows, Version 26.0. Armonk, NY: IBM Corp. The multivariate MCA technique was carried out using R software (R Core Team, 2020, version 3.6.3) with the *FactoMineR* package.

## Results

In our sample, most students scored above the cut-off of both GHQ-12 and USS, with a mean GHQ-12 total score of 6.4 (SD = 2.9) and a mean USS total score of 14.5 (SD = 7.7). These results indicated low levels of psychophysical well-being and a high prevalence of psychological distress. The great majority of students (*n* = 3102) completed the P4 Screener questionnaire, and Table 1 shows the students’ answers to the P4 screener items and the consequent levels of potential suicide risk.

**Table 1:** P4 Screener items and potential risk of suicide categories

		*N*	%
Pre-screening question: Have you had thoughts of actually hurting yourself?	Yes	475	15.3
	No	2627	84.7
*1. Past* suicide attempts	Yes	117	24.6
	No	358	75.4
*2. Plan*	Yes	221	46.5
	No	254	53.5
*3. Probability* of completing suicide	Not at all likely	336	70.7
	Somewhat likely	127	26.7
	Very likely	12	2.5
*4. Preventive* factors	Yes	349	73.5
	No	126	26.5
Potential suicide risk	Did not trigger	2627	84.7
	Minimal	195	6.3
	Lower	62	2.0
	Higher	218	7.0

The most cited types of plan included cutting themselves (32.7%), medication/drug overdose (11.9%), and defenestration (7.0%). The most cited preventive factors were family, partner, and friends (57.0%), and hope in the future (26.9%).

Table 2 shows the association of P4 Screener levels of potential suicide risk with the socio-demographic, academic, and clinical characteristics of the sample.

**Table 2:** Association among P4 Screener levels of suicide risk and socio-demographic, academic, and clinical variables

Variables	Categories	*N*	Potential suicide risk (%)				Chi-square	*p*
			Did not trigger	Minimal	Lower	Higher		
Gender	Male	1282	85.4	6.0	1.7	6.9	1.209	.751
	Female	1815	84.4	6.4	2.2	7.1		
Citizenship	Italian	2941	84.6	6.4	2.0	7.0	.549	.908
	Other	161	85.7	5.0	1.9	7.5		
Religion	Atheist/agnostic	1045	79.8	8.0	2.7	9.5	33.233	** < .001**
	Religious (any religion)	2057	87.6	5.2	1.6	5.6		
Sexual orientation	Heterosexual	2833	86.3	5.4	1.8	6.4	71.391	** < .001**
	Other	219	66.9	15.7	3.5	13.8		
Major	Medicine	1191	82.8	8.1	2.4	6.6	13.711	**.003**
	Other	1911	85.9	5.1	1.7	7.3		
University registration	Regular academic year	2485	85.5	6.0	1.9	6.6	6.869	.076
	Supplementary academic year	617	81.4	7.3	2.6	8.8		
Mean grades	Low (18–22)	453	83.0	4.2	3.8	9.1	16.820	**.010**
	Medium (22–26)	1548	85.1	6.3	1.6	7.0		
	High (26–30)	1101	84.8	7.1	1.8	6.3		
GHQ-12 score	Above cut-off score	2572	83.1	6.6	2.2	8.1	33.872	** < .001**
	Below cut-off score	530	92.3	4.9	0.9	1.9		
USS score	Above cut-off score	1581	77.0	9.2	3.2	10.6	133.254	** < .001**
	Below cut-off score	1296	92.7	3.4	0.6	3.2		
Risky alcohol use	Low risk	2198	85.9	6.4	1.8	5.9	35.320	** < .001**
	Medium/high risk	617	77.1	7.8	2.8	12.3		
Risky use for psychoactive substances	Low risk	2382	86.4	6.3	1.6	5.8	76.533	** < .001**
	Medium/high risk	433	70.9	9.2	4.4	15.5		
Self-rated physical health status	Excellent	429	93.0	3.5	0.7	2.8	68.973	** < .001**
	Good	1529	86.4	5.6	1.9	6.1		
	Fair	1032	80.0	8.3	2.1	9.5		
	Poor	112	72.3	7.1	7.1	13.4		

*Note:* Significant (bold) *p*-values are reported. GHQ-12 cut-off score = 3; USS cut-off score = 13; categories for risky alcohol use = 0–10 score (low risk), above 10 (medium/high risk); categories for risky use for psychoactive substances = 0–3 score (low risk), above 3 (medium/high risk)

With the exception of gender, citizenship, and university registration (being registered on a regular academic year versus being on a supplementary year of schooling), all categorical variables included in the analysis were associated with potential suicide risk as measured by the P4 Screener. More specifically, the associations between potential suicide risk and religion, sexual orientation, major, mean grades, GHQ-12 score, USS score, risky alcohol and substance use, and self-rated physical health status were all statistically significant. Instead, the association between potential suicide risk and age was not statistically significant (*F* = 0.852, *p* = 0.465).

**Fig. 1:**
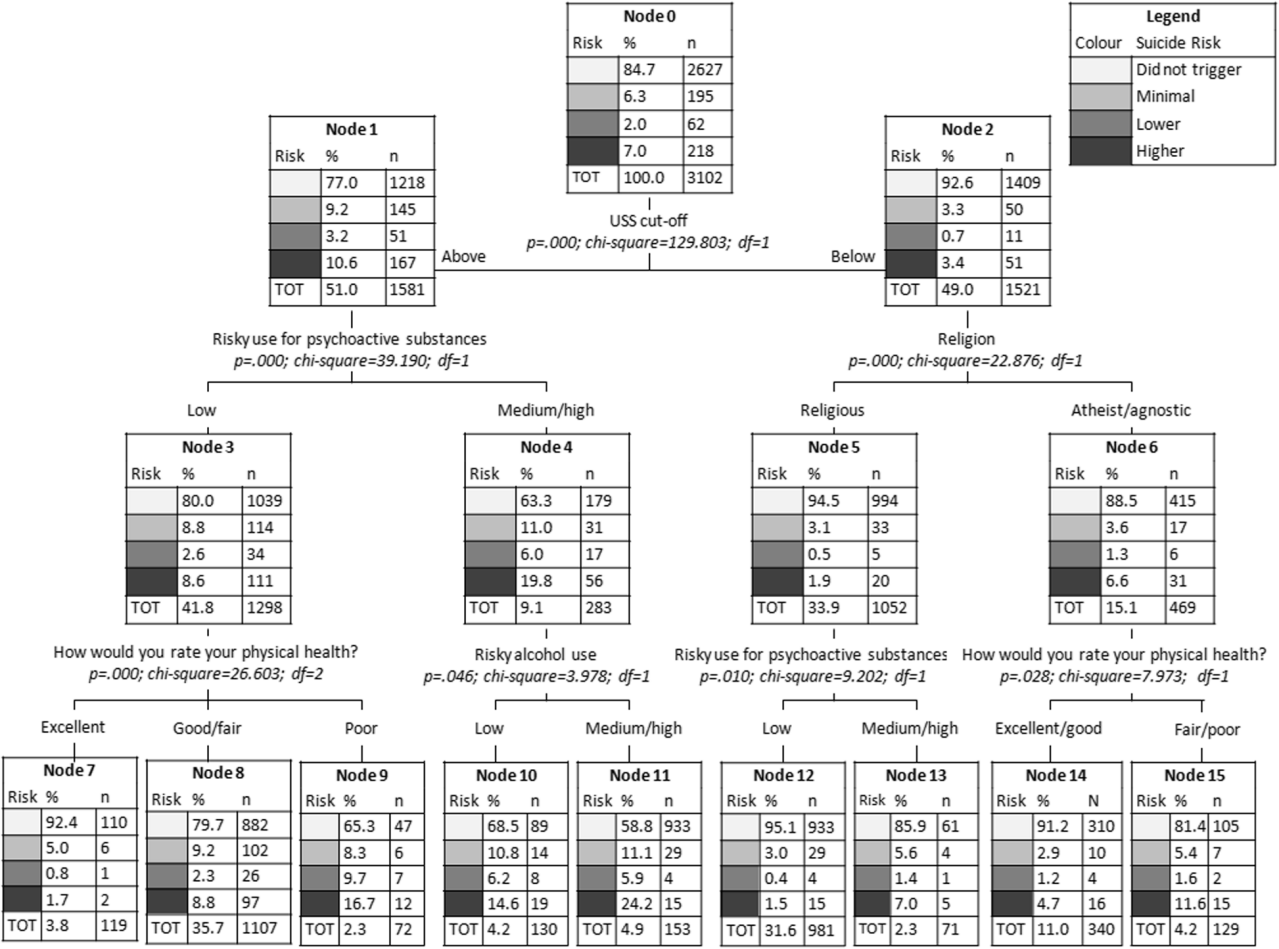
Classification tree for P4 Screener categories: Did not trigger, Minimal, Lower, and Higher

**Fig. 2:**
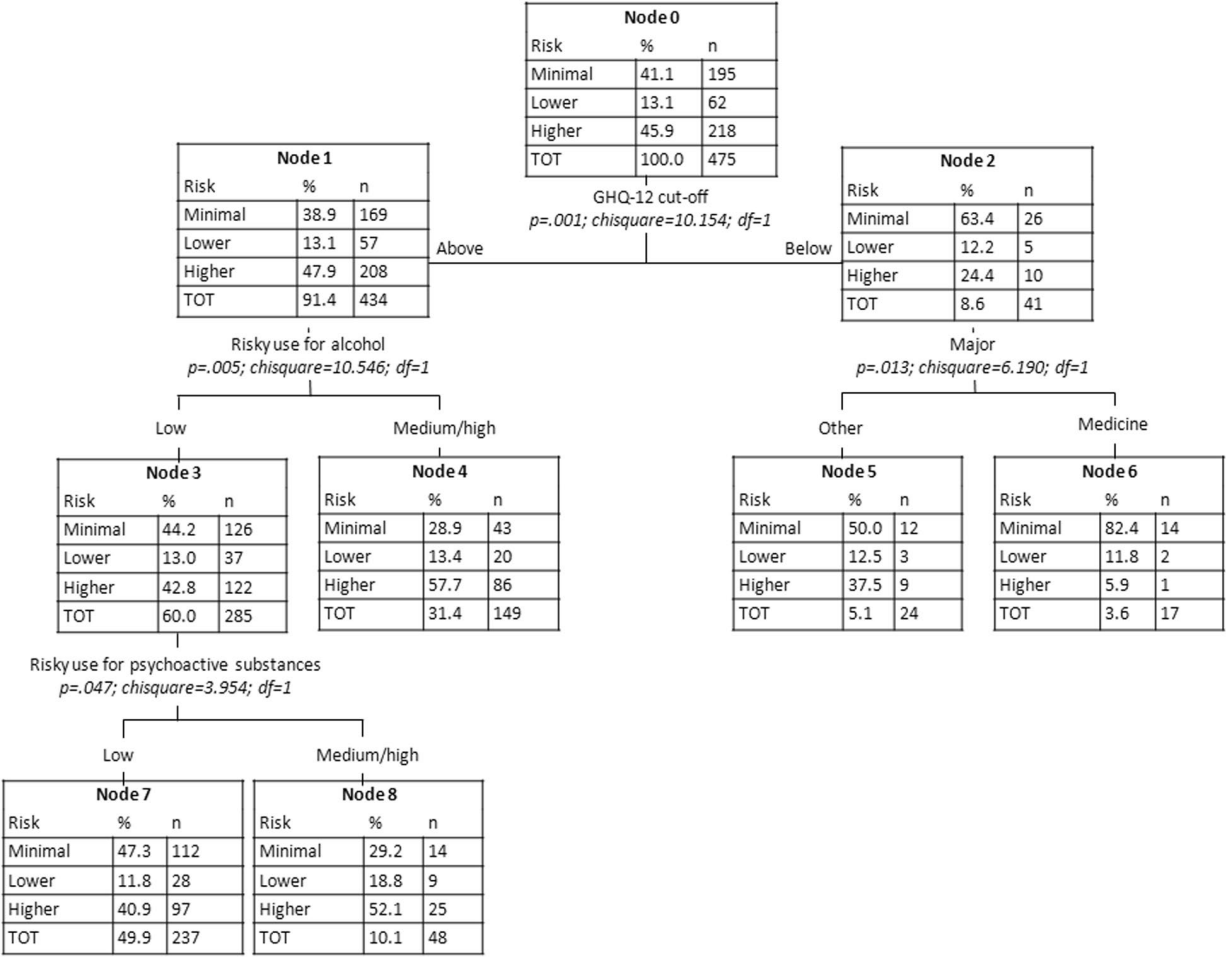
Classification tree for P4 Screener categories: Minimal, Lower, and Higher

Figure 1 shows the CT applied to the P4 Screener with all the significantly associated variables reported in Table 2. The best CT (in terms of predictive accuracy estimated by the confusion matrix with percentage of correct classification) was the one resulting from the Exhaustive CHAID growing method, which is a modification to CHAID that examines all possible splits for each predictor (Bigss et al., 1991). The variable that most discriminated between levels of potential suicide risk was the USS score, dichotomized in “below” and “above” cut-off (Node 1 and Node 2). The CT output revealed that among students scoring below the USS cut-off, a very high percentage were placed in the “did not trigger” category of suicide risk. Among those students scoring below the USS cut-off, individuals who declared they practiced religion and also had a low-risk use of psychoactive drugs were almost entirely (> 95%) in the “did not trigger” category. In this sense, the CT was able to discriminate well between students in the “did not trigger” category and those with at least a minimal potential risk of suicide. Indeed, the confusion matrix showed 100% correct classification for the “did not trigger” category (while the other categories had 0% of correct classification), with an overall percentage of correct classification of 84.7% (see Additional file 1: Tables S1 and S2). In Fig. 2, we therefore investigated the discrimination capability of variables within the subgroup of students (*n* = 475) with potential risk of suicide (minimal, lower, or higher). This analysis showed that students scoring above the GHQ-12 cut-off with a medium/high-risk use of both alcohol and at least one psychoactive substance had over a 50% chance of belonging to the “high risk” group. On the contrary, those scoring below the GHQ-12 cut-off and who were enrolled in a medicine major showed more than an 80% chance of belonging to the “minimal risk” group.

**Fig. 3:**
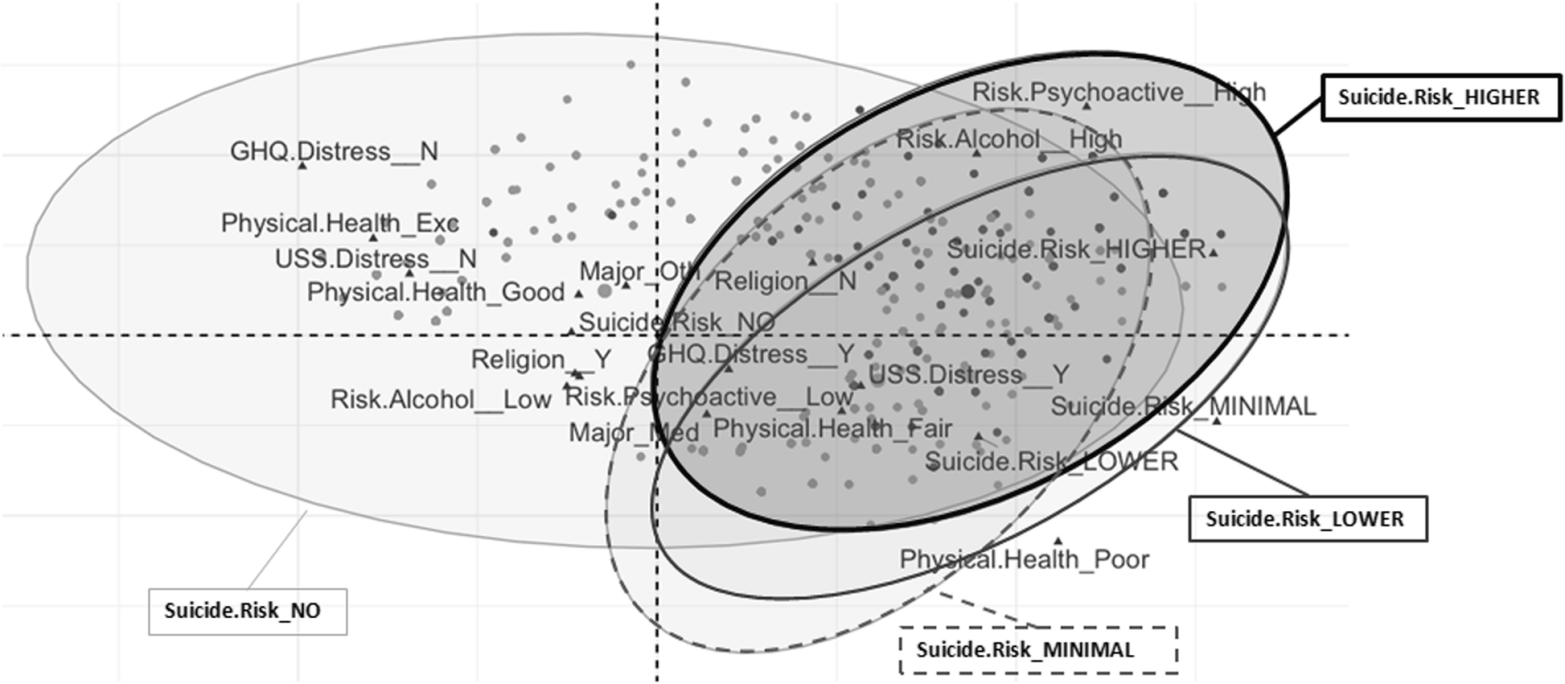
Multiple correspondence analysis biplot for P4 Screener categories: Did not trigger, Minimal, Lower, and Higher. Note: Suicide Risk_NO: “Did not trigger” category. The overlap of the three ellipses with minimal, lower, and higher risk indicates the strict relation among students which presents any level of suicide risk. Differently, the students in “Did not trigger” category constitute a quite separate subgroup of students characterized by “GHQ distress_NO,” “USS distress_NO,” “Physical Health_ Excellent/ Good,” “Risk_Alcohol_Low,” and “Religion_Yes”

**Fig. 4:**
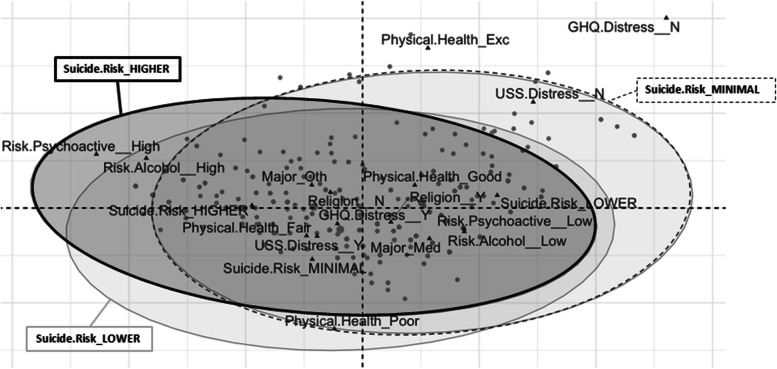
Multiple correspondence analysis biplot for P4 Screener categories: Minimal, Lower, and Higher (*n* = 475). Note: Suicide Risk_NO: “Did not trigger” category. The overlap of the three ellipses indicates the strict relation among students that present any level of suicide risk. However, the minimal risk of suicide group appears more related (close) to the categories “USS distress_NO,” while the higher risk group is associated (close) to the categories of high risk for psychoactive substance and high risk of alcohol use (“Risk Psychoactive/Risk Alcohol_High”)

The MCA confirmed the CT risk profiles. The first MCA (Fig. 3) included all four P4 Screener categories and showed how the profile of students in the “did not trigger” category was characterized by the following: both GHQ-12 and USS scores below the cut-off, excellent or good physical health, a low-risk alcohol use, and practicing a religion. The ellipses of the other three levels of suicide risk overlapped. The second MCA (Fig. 4) only included students (*n* = 475) in the three levels of potential suicide risk (minimal, lower, and higher). Although the three ellipses also overlapped in this case, the profile of students with minimal potential suicide risk was characterized by a USS score of below the cut-off, while the profile of students with higher potential suicide risk was characterized by a medium/high-risk use of both alcohol and psychoactive substances.

## Discussion

Suicidal ideation and attempts are common in young people and university students, and this represents an urgent public health issue. Interestingly, our results indicated lower rates of suicidal thoughts (15%, lifetime) and behavior (3.8%, lifetime) compared to other studies. In a prevalent Scottish study of 18- to 34-year-olds, 11% of participants reported a suicide attempt at some stage in their lives (O’Connor et al., 2018). Considering the specific population of college students, Eskin et al. (2016) found that almost 29% of the participants among their international samples reported suicidal thoughts and 7% had attempted suicide, while in the study conducted by Sivertsen et al. (2019) among Norwegian college students, lifetime suicidal thoughts were reported by 21% of participants. This difference may be explained by considering that, among European Union states, Italy has one of the lowest suicide rates (six per 100,000 inhabitants), with an average European suicide rate of 11 per 100,000 inhabitants (European Commission, 2018).

Identifying risk and protective factors is a key component of suicide prevention strategies. However, existing research showed that these factors are weak and inaccurate predictors of suicidal thoughts and behaviors. Single risk factors are not enough to predict suicide; instead, research should focus on complex combinations of predictors and develop tailored algorithms for specific populations (Franklin et al., 2017). Traditional statistical methods struggle to capture the intricate relationships between suicide risk factors, while machine learning may offer a powerful alternative, capable of modeling these complex interactions.

In our study, the results of chi-square and ANOVA tests supported the hypothesis that non-heterosexual orientation, lower mean grades, significant psychological distress, and a medium/high-risk use of alcohol and other psychoactive substances were risk factors for potential suicide risk in college students. On the contrary, practicing a religion appeared to be a possible protective factor, again in line with previous research (Assari, 2018). We also recorded some results in contrast with previous studies: with regard to university registration, we cannot confirm previous research (Uchida & Uchida, 2017) claiming that extra years of schooling represented a risk factor for suicide, but again we may hypothesize that the different cultural background of this Japanese study may explain the different impact of not being registered in a regular academic year, which in our sample was pretty common (19.8% of all students). Moreover, our results showed that gender was not associated with potential suicide risk, while the World Health Organization has confirmed that, worldwide, men are nearly twice as likely to die of suicide than women (World Health Organization, 2021). Previous studies on college students, however, have shown mixed results (Mackenzie et al., 2011; Mortier et al., 2018; Uchida & Uchida, 2017). This may be explained by considering that we measured potential suicide risk and not actual suicide attempts, and this difference perhaps suggests the need for further research into pathways to describe how male and female students, with similar levels of potential risk, may reach different rates of suicidal behavior.

The subsequent CTs and MCA techniques provided valuable insights beyond those from single predictors providing a definition of subgroups of students with different levels of suicide risk based on specific predictor values/cut-offs. The CT including the 475 students presenting at least a minimal suicide risk showed that psychological distress, use of alcohol, and use of other psychoactive substances were the best predictors of suicide risk. These results were expected, considering the number of studies supporting the definition of these factors as important risk factors (Arria et al., 2009; Assari, 2018; Eskin et al., 2016; Garlow et al., 2008; Shen et al., 2020) and considering that the use of psychoactive substances and alcohol represents a common coping strategy among people with poor mental health (Armeli et al., 2014; Holahan et al., 2001). In addition, both CT and MCA allowed to define specific profiles: students having a significant level of psychological distress, with a medium/high-risk use of both alcohol and at least one psychoactive substance had over a 50% chance of belonging to the “high risk” group. The identification of this risk profile can be highly relevant for university mental health services in promoting mental well-being, suggesting a specific student subpopulation that requires prioritized preventive interventions regarding suicide risk. Additionally, this highlights the importance of targeted preventive interventions that target not only risky alcohol and substance use but also well-being factors. Such a comprehensive approach would optimize resource allocation by focusing on students most at risk and tackling contributing factors simultaneously. Interestingly, in the same CT, results found that medical students with sufficient well-being had an over 80% chance of falling into the “minimal risk” group. These findings contrast with research by Uchida and Uchida (2017) on medical students in Japan. While the specific reasons for this difference are unclear, it is possible that cultural background plays a partial role. Additionally, medical students might have better mental health literacy due to their studies, and mental health literacy has been recognized as a protective factor against suicidality (Goldney et al., 2002; Lindow et al., 2020).

On the other hand, the CT and the MCA performed on the whole sample allowed us to shed some light on students who have not had any thoughts of actually harming themselves. Indeed, in the CT, practicing a religion was a feature that identified the “did not trigger” subgroup (describing students with no thoughts of hurting themselves), therefore confirming this as a protective characteristic for suicide risk, as suggested by (Assari 2018). More specifically, among students not presenting significant levels of academic distress, individuals who declared they practiced a religion and also had a low-risk use of psychoactive drugs were almost entirely in the “did not trigger” group. The student profile defined by the “did not trigger” subgroup offers valuable insights for preventive interventions. It highlights the effectiveness of promoting low academic distress, alongside attention to spiritual well-being and a healthy lifestyle that fosters good physical health, in protecting students from suicidal risk. The MCA analysis further supports this notion, as the profile of the “did not trigger” students was characterized by practicing a religion, low level of distress, excellent or good physical health, and low-risk alcohol use. Religion as a protective factor may also help to explain why Italy has such a low suicide rate, as it has one of the highest rates of people practicing a religion in Europe (European Commission, 2010), and also considering that Catholicism is the most common religion in the country, which has a strong position against suicide.

This study has potential limitations. Firstly, although the sample was very large, it may not provide the best generalization, because it came from a single Italian university. This bias should also be considered in light of the relatively low response rate, although available data on online surveys among university student populations show variable response rates (Kim et al., 2018; Schwenk et al., 2010). As suggested by previous research (Edwards et al., 2009; Sammut et al., 2021), the odds of response to a web-survey can greatly vary considering the different design and characteristics of the web-survey itself, and response rates can be increased with a wide range of strategies including the use of non-monetary incentives, shorter and personalized e-questionnaires, email invitations, and reminders. We did our best to maximize the response rate using several strategies suggested by Edwards et al. (2009); however, we could not include incentives, and the length of our online survey was not very short because we aimed at collecting a multidimensional set of data on students’ mental health and wellbeing. For such reasons, further studies including more representative samples and cross-cultural comparisons are needed to improve the generalizability of results. Secondly, we considered a lot of possible predictors, but we omitted some variables (such as family income, social support or coping strategies). This may have led to some potentially relevant predictors being omitted; further studies including a wider range of variables would be desirable. Having said that, we decided to limit the number of questions in order to avoid a lower response rate, often caused by longer online surveys (Edwards et al., 2009). Thirdly, to maximize online survey participation, we prioritized self-administered and brief assessment tools. However, these instruments lacked validation specifically for the Italian college student population. While standardized measures validated for this specific population might be preferable, we were unable to identify alternatives that combined self-administration, brevity, online compatibility, and validation in this specific context. Nevertheless, we examined the Cronbach’s alpha for each instrument in our sample, revealing values similar to those reported in the original validation studies and in other Italian studies, when available. Finally, it is important to note that machine learning tools are not without their limitations. First of all, they can still be susceptible to the same problems that have hindered the adoption of previous suicide prediction methods, including the problem of the low base rate of suicidality (McHugh & Large, 2020). Furthermore, concerns have been raised that the focus on machine learning might blur the crucial distinction between statistical validity and clinical utility, especially considering the potential challenges faced by clinicians and policymakers in comprehending the employed statistical methods (McHugh & Large, 2020). Therefore, while machine learning tools have the potential to significantly impact the prediction of suicidality, caution is warranted in the application of these techniques and the interpretation of their results. However, our choice to apply two different machine learning techniques on a sufficiently large dataset analyzed following well-established hypotheses allowed us to address adequately their limitations.

## Conclusions

This study was conducted on a large sample of Italian college students and it identified predictors and student profiles associated with suicide risk through a machine learning approach. Our findings may be useful for researchers, health professionals, and policymakers seeking to identify the predictors of suicide risk in the college population, in order to take action to prevent suicide. Developing an efficient model able to assess suicide risk would be a crucial step forward for suicide prevention. Our approach highlighted the importance of considering multidimensional variables including mental health measures, academic factors, and socio-demographic characteristics, by leveraging machine learning algorithms in order to capture their intricate relationships and establish risk profiles for college students. This might help the development of targeted preventive measures. Indeed, effective prevention programs need to address both risk and protective factors, as well as being tailored to specific categories of risk. From this point of view, it would be desirable to improve the academic counseling services in order to intercept more vulnerable students. College counseling can represent a key front-line service in early detecting sub-threshold symptoms related to mental distress among college students. A “one size fits all” approach for the prevention of suicide behaviors is unlikely to be effective for everyone, and a preliminary assessment of the level of risk may greatly contribute to the implementation of a personalized approach*.*

### Supplementary Information

Additional file1: Table S1. Table S2

## Abbreviations

GHQ-12: General Health Questionnaire (12 items version)
USS: University Stress Scale
ASSIST: World Health Organization’s Alcohol, Smoking and Substance Involvement Screening Test v.3
SD: Standard deviation
CT: Classification tree
MCA: Multiple correspondence analysis
