# Supplementary material for: Potential suicide risk among the college student population: machine learning approaches for identifying predictors and different students’ risk profiles
Source: Psicol Reflex Crit. 2024 May 17;37:19. doi: 10.1186/s41155-024-00301-6 (PMC11101401; doi:10.1186/s41155-024-00301-6)
Supplement: Supplementary file 1 — Additional file1: Table S1. Table S2 [file 41155_2024_301_MOESM1_ESM.pdf]

Supplemental Online Material

Table S1 Confusion Matrix of the CT for P4 Screener categories: Did not trigger, Minimal, Lower and Higher

Observed	Predicted				
	Did not trigger	Minimal	Lower	Higher	Correct percentage
Did not trigger	2627	0	0	0	100,0%
Minimal	195	0	0	0	0,0%
Lower	62	0	0	0	0,0%
Higher	218	0	0	0	0,0%
Total percentage	100,0%	0,0%	0,0%	0,0%	84,7%

Table S2 Confusion Matrix of the CT for P4 Screener categories: Minimal, Lower and Higher

Observed	Predicted			
	Minimal	Lower	Higher	Correct percentage
Minimal	138	0	57	70,8%
Lower	33	0	29	0,0%
Higher	107	0	111	50,9%
Total percentage	58,5%	0,0%	41,5%	52,4%
